# Ovarian Cystic Lymphangioma

**DOI:** 10.7759/cureus.54983

**Published:** 2024-02-26

**Authors:** Akanksha Sharma, Shivanjali Raghuvanshi, Manish Kumar, Nisha Singh, Nancy Gupta, Arina Alam, Rameez Uddin Nayak

**Affiliations:** 1 Pathology, King George's Medical University, Lucknow, IND; 2 Obstetrics and Gynaecology, King George's Medical University, Lucknow, IND; 3 Pathology, S.N. Medical College, Agra, IND

**Keywords:** laparotomy, oophorectomy, malignancy, ovary, lymphangioma

## Abstract

Lymphangioma are benign, slow-growing and rare lymphatic tumors, which may emerge at any location in the body with ovary being a very rare location. Axillary region and neck are the most common sites, while retroperitoneum and mesentery account for <1%. We present a case of a young female of 33 years who had symptomatic pelvic mass and was presented with a complaint of lower abdominal pain of six-month duration and weight loss. Investigation revealed an oval-shaped complex cystic density lesion in the right adnexal region, which was likely neoplastic. Elective laparotomy with right ovarian cystectomy was performed. Histopathological examination revealed ovarian lymphangioma.

## Introduction

Lymphangiomas are tumors of the lymphatic system that are slow-growing and benign and consist of cystic cavities that are multiple and surrounded by endothelial cells that are single-layered, bland, and flattened. These spaces are interspersed by septa of fibro-collagenous tissue. Lymphangioma may occur at any location within the body. In young children, they mostly occur in the axilla, neck, and scalp region, whereas in adults, intra-abdominal location is predominant [1]. According to the size of lymphatic vessels, lymphoma can be categorized as cavernous, cystic and capillary and are filled with either chylous or serous fluid [2].

Ovarian lymphangioma is an extremely rare condition which is mostly encountered incidentally and is usually asymptomatic for a long time. In a few cases, they grow large enough and give symptoms of lower abdominal pain [3]. They present with nonspecific radiological and clinical findings and sometimes they are mistaken with malignant tumors. Hence histopathological examination is mandatory for definite diagnosis. We present a case of a young female of 33 years who was diagnosed with cystic lymphangioma ovary that was successfully operated.

## Case presentation

A 33-year-old female presented to our hospital with an infertility problem for two years. She complained of lower abdominal pain and dysfunctional uterine bleeding for a period of six months. There was no vomiting, diarrhea, nausea, or weight loss. She did not have bloody stool, constipation, hematuria or urinary frequency. She had no past medical history of radiation exposure, cancer, or trauma. Medical and family history was non-contributory. Her routine hematological and biochemical profiles including full blood count and serum electrolytes were within normal range. Upon physical examination, lower abdominal pain was detected via abdominal palpation. On per speculum examination, cervix and vagina were healthy. There was no discharge. On per vaginal examination, right forniceal fullness was noted and left fornix was clear. Contrast-enhanced computed tomography of abdomen revealed oval-shaped, complex cystic density lesion measuring 6.6x4.5x4 cm in right adnexal region. It showed enhancing components and septations as well as calcific foci. Right ovary was not visualized separately. Left ovary was normally visualized. Differential diagnosis of ovarian neoplasia and inflammatory tubo-ovarian mass was made. Transvaginal sonography with color Doppler showed massively enlarged right ovary having internal vascularity and showing mildly heterogeneous echotexture with thickened stroma and peripherally displaced follicles. Features were suggestive of partial torsion and exophytic ovarian hemorrhagic cyst. Because her radiological findings indicated malignancy, she was investigated for serum tumor markers. Carcinoembryonic antigen, CA 19-9, and alfafetoprotein were within normal limits. There was no evidence of lymphadenopathy anywhere in the body.

The precise reason behind the enlargement of the right ovary could not be determined. Thus, the decision was made to move forward with an exploratory laparotomy. Combined spinal-epidural anesthesia was given. 3 ml of 0.5% injection bupivacaine was given for subarachnoid block. An enlarged right ovary with a white and gray cystic lobulated mass with internal septation and several cystic regions filled with milky fluid and cheese-like substance was discovered during exploration. Cystic content was drained. Left ovary and uterus were unremarkable and small quantity of chylous milky fluid was found in the pouch of Douglas. There was no implantation on peritoneum. The tubo-ovarian and infundibulopelvic ligaments on the right side were clamped, cut and tied and salpingo-oophorectomy was done on the right side, and the tumor was removed. Patient’s post-operative course was uneventful and unremarkable. 

**Figure 1:**
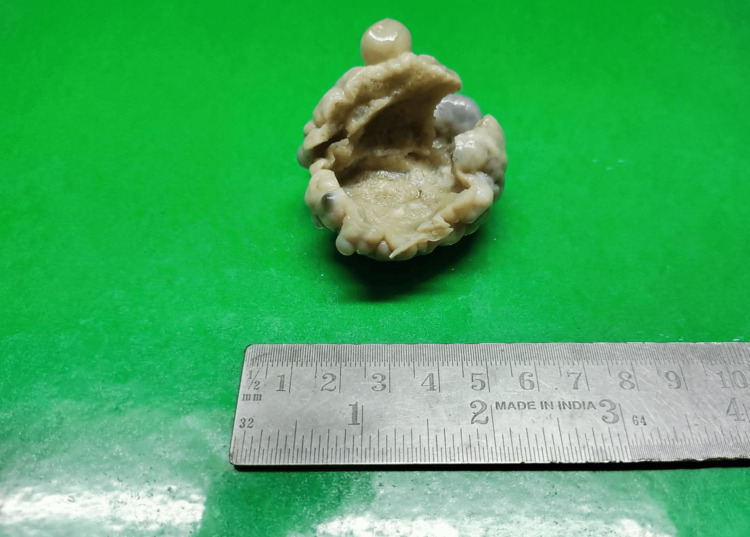
Gross photograph of cut surface of ovary showing oval, soft, cystic space measuring 4x3x2 cm.

**Figure 2:**
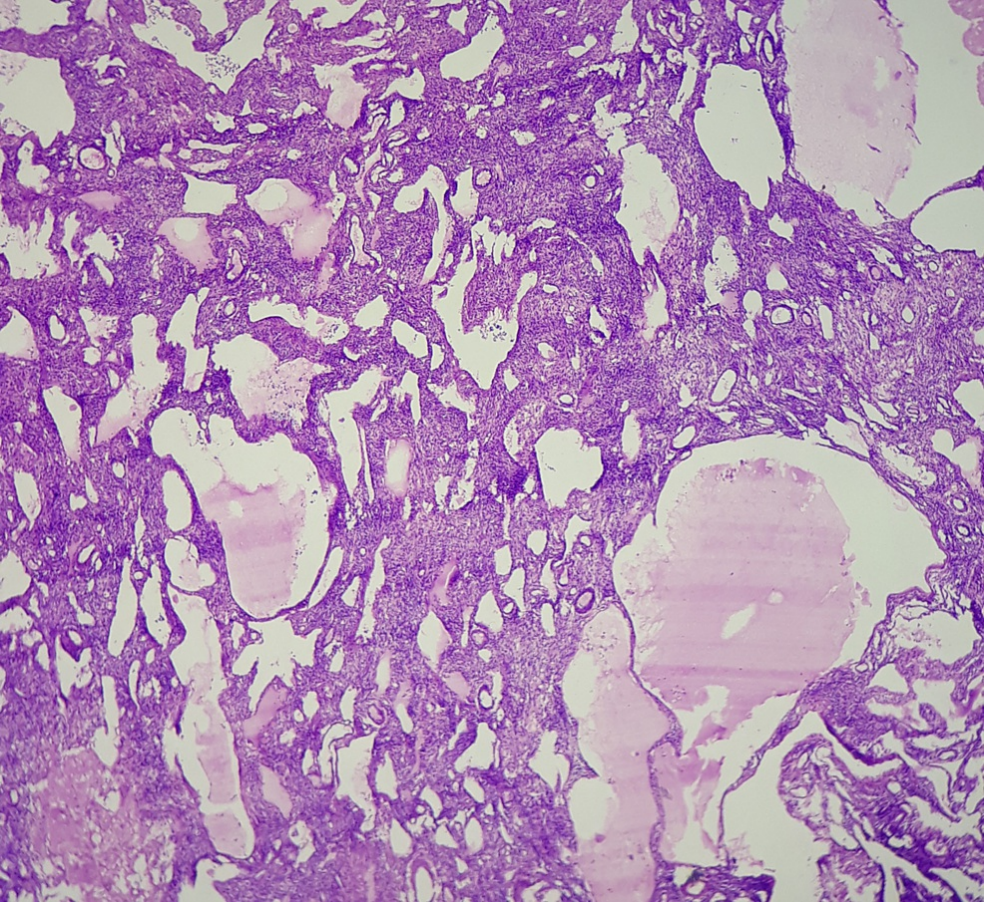
H&E x100 Ovarian stroma infiltrated by multiple cystic spaces

**Figure 3:**
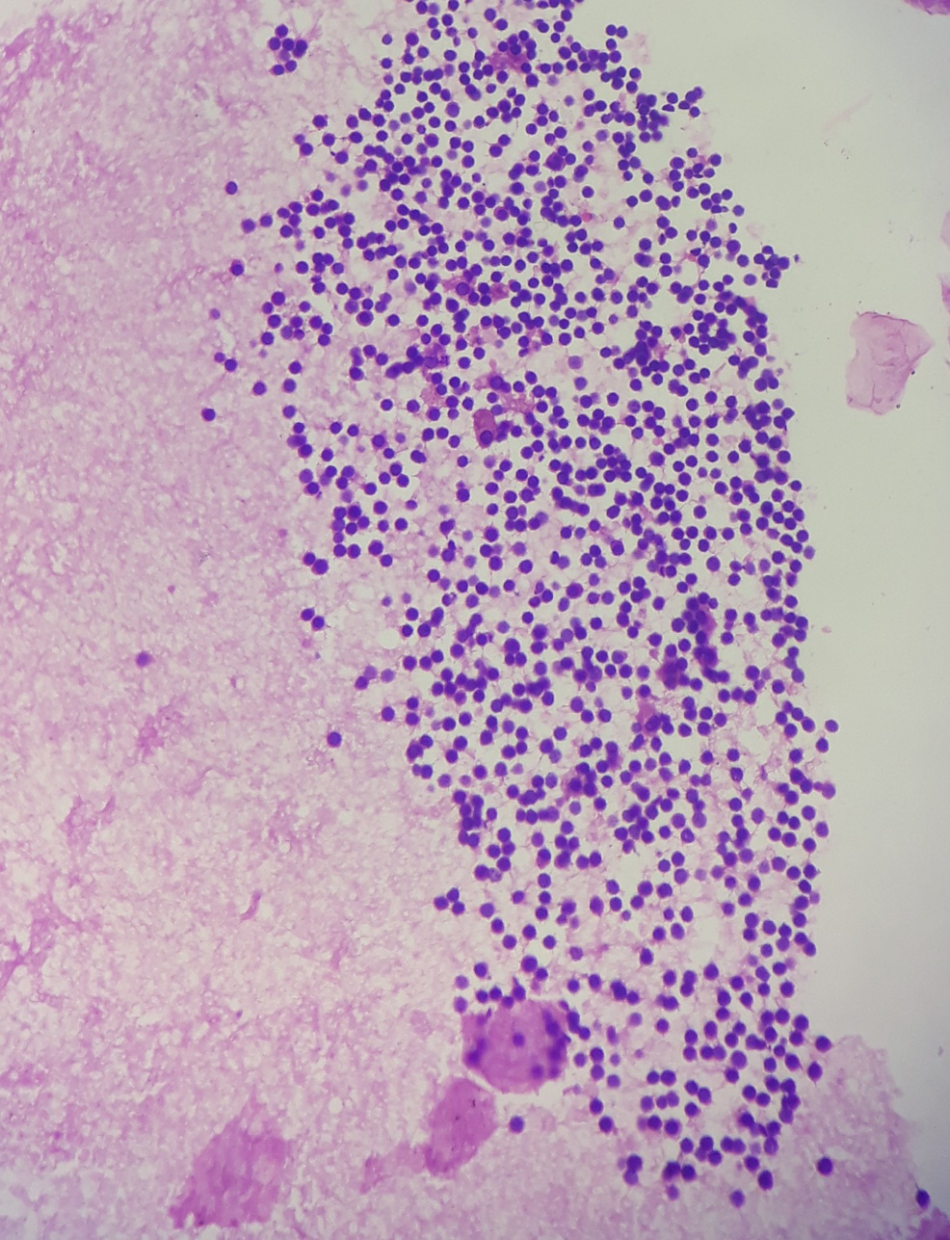
H&E x200 Lumen filled with lymph fluid and lymphocytes

Gross examination revealed mass from the right ovary which measured 4x3x2 cm (Figure 1). Cut surface showed multiple cystic spaces of varying size which contained straw color fluid in the lumen. Histopathological analysis of cystic mass from the right ovary showed dilated and multiple vascular spaces of variable size which were surrounded by endothelial cells which were single layered, bland and flattened. These cells were having benign morphology. Hyperchromasia and atypia were absent. There was no evidence of necrosis. Mitosis was absent. Cystic spaces were intervened by septa of fibrocollagenous tissue (Figures 2, 3). Diagnosis of lymphangioma was made. The patient was thereafter monitored. Prognosis and evaluation was good and following a six-month follow-up period including clinical and ultrasonography monitoring, no recurrence was observed.

## Discussion

The lymphatic system comprises unidirectional lymphatic vascular channels that transport lymph toward the subclavian vein [4]. Lymphangiomas are rare and benign lesions that most often occur in the head, axillary region and neck. Visceral lymphangiomas are very rare and mostly occur in the mesentery and intestine. Ovarian lymphangioma was first described by Kroemer in 1908 [5]. Only 21 ovarian lymphangioma instances have been reported in the literature to date, out of which two cases were of neonatal age group. One of them was presented with torsion of ovary in utero [6]. Because it is an extremely rare entity, its pathogenesis is still not very clear. Few authors suggest that during embryonic development, there is sequestration of lymphatic tissue and proposed lymphangioma to be a hamartomatous process. Others suggest that they are true neoplasms. In a case report, the patient had symptoms of chronic follicular salpingitis and later presented with ovarian lymphangioma. The underlying cause of lymphangioma was found to be a disturbance in the drainage of the lymphatic system [2]. Another case report found that lymphangioma develops in the ovary as a result of radiation exposure for treatment of Wilms tumor [7]. Ovarian lymphangioma in menopausal women was reported by Akyildiz et al. and they found a huge leiomyoma of 35 cm diameter was obstructing the lymphatic circulation [8].

Radiation therapy, trauma, mechanical pressure, lymph node degeneration, and inflammatory and genetic factors can be a predisposing factor for acquired lymphangiectasia leading to lymphangioma. In our case, the patient did not have any such complaints, therefore hamartomatous lesions were suggested. In the majority of cases, ovarian lymphangioma remains asymptomatic and presents as a slow-growing tumor. After excision, they are incidentally identified on histopathological examination. Lymphangioma can present as an asymptomatic mass or acute abdomen. The position and size of lymphangioma are the main determining factors that play an important role in presentation. One of the most common initial signs of pelvic and intra-abdominal lymphangiomas is abdominal distension and pain. Ovarian lymphangiomas may give rise to complications like collapse, volvulus, signs of compression, secondary infection, and bowel obstruction [9].

In literature, to date, only one case of bilateral ovarian lymphangioma has been documented. Mostly these are unilateral [10]. Sometimes the presentation is very vague. A case has been reported in which the patient presents with chyluria and chylous ascites [11]. In very few cases ovarian lymphangioma increases in size and compresses the adjacent organs. A case of large-size ovarian lymphangioma has been reported. They are typically seen in the parenchyma or on the surface of the ovary [12]. Lymphangioma comprises multiple, thin-walled vascular spaces which are closely packed and lined by endothelial cells. Pale and eosinophilic material was present in the lumen, which is called lymph. Along with it abundant lymphocytes are also present. Lymphocytic aggregates are also present in the stroma. The primary histological differential diagnosis of ovarian lymphangioma includes hemangioma, secondary lymph channel dilatation, teratoma with a significant vascular component, and adenomatoid tumor. Positive periodic acid-Schiff (PAS) and Alcian blue staining of adenomatoid tumors aids in their differentiation from lymphangiomas [13].

The main differential diagnoses for ovarian lymphangioma are hemangioma and adenomatoid tumor. The vascular channels which are thin-walled and surrounded by endothelial cells which are flat and benign and the lumen filled with eosinophilic material called lymph helps to arise at diagnosis of lymphangioma [14]. The differential diagnosis considered in our case was hemangioma. Lymphangioma and hemangioma are histologically similar, but vascular lumen is filled with more red cells in case of hemangioma, and lined by endothelial cells which are continuous. 

Lymphangioma are converted into malignancy with extreme rarity. Rice et al. have reported malignant transformation in lymphangioma. Rice et al. described a case of lymphangioma of ovary which was converted into malignancy with involvement of the opposite ovary. These lesions were also metastasized to the liver and peritoneum was diffusely involved. The lesion recurred within six months [15]. Aristizabel et al. described twice recurrence of lymphangioma occurring within a time duration of two years. The Recurrence lesion was histologically identical to the previous lesion. Patients ultimately require radiation therapy for management [16]. Lymphangiomas are also associated with some complications like rupture, volvulus, intestinal obstruction, secondary infection and compression of adjacent structures due to mass effect [17]. For emergency cases, cysts can be aspirated and sclerosant agents may be injected, but in elective cases these modalities are not recommended because there are high chances of recurrence. Surgery is the preferred route of management, including both laparoscopy and laparotomy. The imaging method which is most preferred in adnexal masses is pelvic ultrasonography. Magnetic resonance imaging or computed tomography may distinguish straw color fluid from blood and pus, differentiate benign lesions from malignant and help to identify anatomical relationships with other structures [18]. Many differentials should be considered in ovarian cysts like paraovarian cyst, obstruction of fallopian tube, inclusion cyst of peritoneum and appendiceal mucocele.

Surgical management of ovarian lymphangioma is required because there are chances of recurrence and it does not regress spontaneously. Post-operative complications are few and include torsion, bleeding and abscess. In some cases peritonitis may occur [19]. For treatment, accepted routes of surgery are both laparoscopy and laparotomy. Only a few case reports are available in the literature that describe laparoscopic treatment of an ovarian lymphangioma, whereas several reports are available describing successful open resection of intra-abdominal lymphangioma [20].

## Conclusions

In the case of intra-abdominal masses complete preoperative diagnostic investigation is needed to decide appropriate surgical procedure. Magnetic resonance imaging and computed tomography help in preoperative surgical management by giving information regarding the location of the lesion, size of the lesion, and involvement of adjacent organs. This case emphasizes the significance of considering lymphangioma among provisional diagnoses of multicystic lesions of the ovary. A confirmatory diagnosis is a histopathological examination. 
